# Targeting VISTA for immunomodulation in sepsis: mechanisms and therapeutic potentials

**DOI:** 10.3389/fimmu.2026.1850679

**Published:** 2026-05-29

**Authors:** Baoji Hu, Jiangbin Yan, Wentao Ji, Huixian Wang, Tianzhu Tao, Lulong Bo

**Affiliations:** 1 Department of Anesthesiology, Shanghai Pudong Hospital, Fudan University Pudong Medical Center, Shanghai, China; 2 Faculty of Anesthesiology, Changhai Hospital, Naval Medical University, Shanghai, China; 3 Department of Anesthesiology, Shanghai East Hospital, School of Medicine, Tongji University, Shanghai, China; 4 Department of Anesthesiology, Air Force Medical Center, Beijing, China

**Keywords:** immune checkpoint, immunometabolism, immunosuppression, organ dysfunction, sepsis, targeted therapy, VISTA, VSIR

## Abstract

Sepsis is a life-threatening clinical syndrome defined as acute organ dysfunction caused by a dysregulated host response to infection. At the core of its pathophysiology lies the aberrant activation and dysregulation of the host immune system. The V-domain Ig suppressor of T cell activation (VISTA) is a dual-function immune checkpoint molecule that acts as both a receptor and a ligand, and exerts a pivotal immunoregulatory role in the pathological process of sepsis. Emerging preclinical and clinical evidence indicates that VISTA exerts stage-specific biphasic effects during the progression of sepsis. In the early hyperinflammatory phase, VISTA signaling potentially alleviates the cytokine storm and preserves the integrity of organ barriers; in contrast, during the subsequent immunosuppressive (immune paralysis) phase, aberrant VISTA upregulation may drive the sustained hyporesponsiveness of T cells and the tolerogenic reprogramming of myeloid cells. In this review, we systematically summarize and critically appraise the dynamic expression profiles of VISTA across different stages of sepsis, and synthesize current evidence regarding the multifaceted mechanisms by which VISTA modulates both excessive inflammatory responses and immunosuppression. We further evaluate preclinical studies investigating VISTA-targeted interventions, with a focus on their impacts on survival outcomes, organ injury, and immune cell function. Collectively, this review highlights the central regulatory role of VISTA in the immunoregulatory network of sepsis, and proposes that precision immunomodulatory strategies targeting VISTA hold significant promise as a novel therapeutic approach for patients with sepsis.

## 1 Introduction

Sepsis and septic shock remain among the most formidable life-threatening syndromes in modern critical care medicine. The Third International Consensus Definitions for Sepsis and Septic Shock (Sepsis-3) define sepsis as acute, life-threatening organ dysfunction caused by a dysregulated host response to infection (1–3). This landmark definition reframes sepsis as a syndrome driven by multifactorial, network-wide perturbations across the immune, metabolic, and microcirculatory systems, rather than a purely hyperinflammatory state (4). While early recognition, timely antimicrobial therapy, goal-directed fluid resuscitation, and organ support have been established as the standard of care for sepsis, data from the Global Burden of Disease Study consistently demonstrate that sepsis remains a leading cause of morbidity and mortality worldwide, with persistently high disease burden in both high-income and low- and middle-income settings (5, 6). This unmet clinical need highlights an urgent demand for novel, host-directed therapeutic strategies that target the core pathophysiological drivers of sepsis. In particular, recent preclinical and clinical evidence has emphasized the promise of immune status-guided precision medicine and immunophenotype-driven interventional trials for this heterogeneous syndrome.

Over the past few decades, anti-inflammatory strategies targeting single pro-inflammatory mediators (including tumor necrosis factor [TNF], interleukin-1 [IL-1], and endotoxin) have failed to demonstrate consistent survival benefits in large-scale randomized controlled trials (7, 8). These neutral findings have prompted a fundamental reassessment of the temporal and spatial heterogeneity of the host immune response in sepsis. Specifically, hyperinflammation and immunosuppression may not only occur sequentially at different disease stages in the same patient, but may also coexist simultaneously within distinct tissue compartments (4, 8, 9). Furthermore, the immune milieu in the peripheral circulation is frequently discordant with that in affected organ tissues, further complicating the assessment of an individual patient’s immune status. Accordingly, sepsis immunotherapy requires dynamic, individualized calibration based on a patient’s immune phenotype, pathogen burden, and pattern of organ dysfunction, an approach that forms the cornerstone of precision immunomodulation for sepsis (10, 11).

In recent years, immune checkpoint molecules have been identified as central regulators bridging physiological immune homeostasis and the pathological immunosuppression that characterizes late-stage sepsis. Accumulating preclinical and clinical evidence demonstrates that multiple immune checkpoints, including programmed cell death protein 1 (PD-1)/programmed cell death ligand 1 (PD-L1), cytotoxic T-lymphocyte-associated protein 4 (CTLA-4), and T-cell immunoglobulin and mucin domain-containing protein 3 (TIM-3), are significantly upregulated in both animal models of sepsis and clinical samples from septic patients (12–15). Importantly, modulation of these pathways (either via blockade or activation) yields divergent, even opposing, outcomes depending on the phase of sepsis and the underlying immune phenotype of the host (16–18). This observation underscores a core principle: sepsis immunotherapy cannot be reduced to non-specific immune enhancement or suppression, but should instead aim to restore and maintain physiological immune homeostasis. Systematic evaluations have further revealed that checkpoint molecule expression patterns and treatment responses to checkpoint modulation vary widely across preclinical models and clinical patient subsets, reinforcing the critical need for biomarker-based patient stratification and rationally designed combination therapeutic approaches (19–21).

Among these emerging immune checkpoints, the V-domain Ig suppressor of T cell activation (VISTA) has garnered substantial research interest due to its unique biological properties. Unlike the PD-1/PD-L1 axis, which is primarily upregulated upon immune activation, VISTA is constitutively and highly expressed on myeloid cells—the primary drivers of both the early hyperinflammatory cascade and late immunosuppressive reprogramming in sepsis—and functions as both a receptor and a ligand to mediate bidirectional immunoregulation. By calibrating the activation threshold of the innate immune system and modulating the balance between immune activation and tolerance, VISTA represents a particularly promising target for host-directed therapy in sepsis (22–24). In this review, we systematically summarize the molecular characteristics of VISTA and its ligand interaction network, synthesize current preclinical and clinical evidence linking dysregulated VISTA signaling to sepsis-associated immune dysfunction, organ injury, and clinical outcomes. While VISTA shows great promise as a therapeutic target, the field is currently limited by a lack of direct clinical evidence linking VISTA expression dynamics to patient outcomes, and that this gap is a major focus of the challenges discussed. We further discuss the therapeutic potential of VISTA-targeted strategies for sepsis, alongside the key challenges and future directions for advancing these interventions into clinical translation.

## 2 Multifaceted and context-dependent immune checkpoint regulator in health and disease

### 2.1 Molecular and structural characteristics of VISTA

VISTA is a well-characterized immunoregulatory molecule belonging to the immunoglobulin (Ig) superfamily, which exerts potent negative regulatory effects on T cell-mediated immune responses. VISTA was initially identified and characterized in murine models, with its function as a non-canonical immune checkpoint in human T cells validated in subsequent mechanistic studies (25, 26). VISTA is classified as a non-canonical member of the B7-CD28 immunoregulatory superfamily, with partial structural and functional homology to other family members (27). It has multiple previously reported aliases, including PD-1H, B7-H5, Dies1, Gi24, DD1α, and C10orf54. In humans and mice, VISTA is encoded by the *VSIR* and *Vsir* genes, respectively.

**Figure 1:**
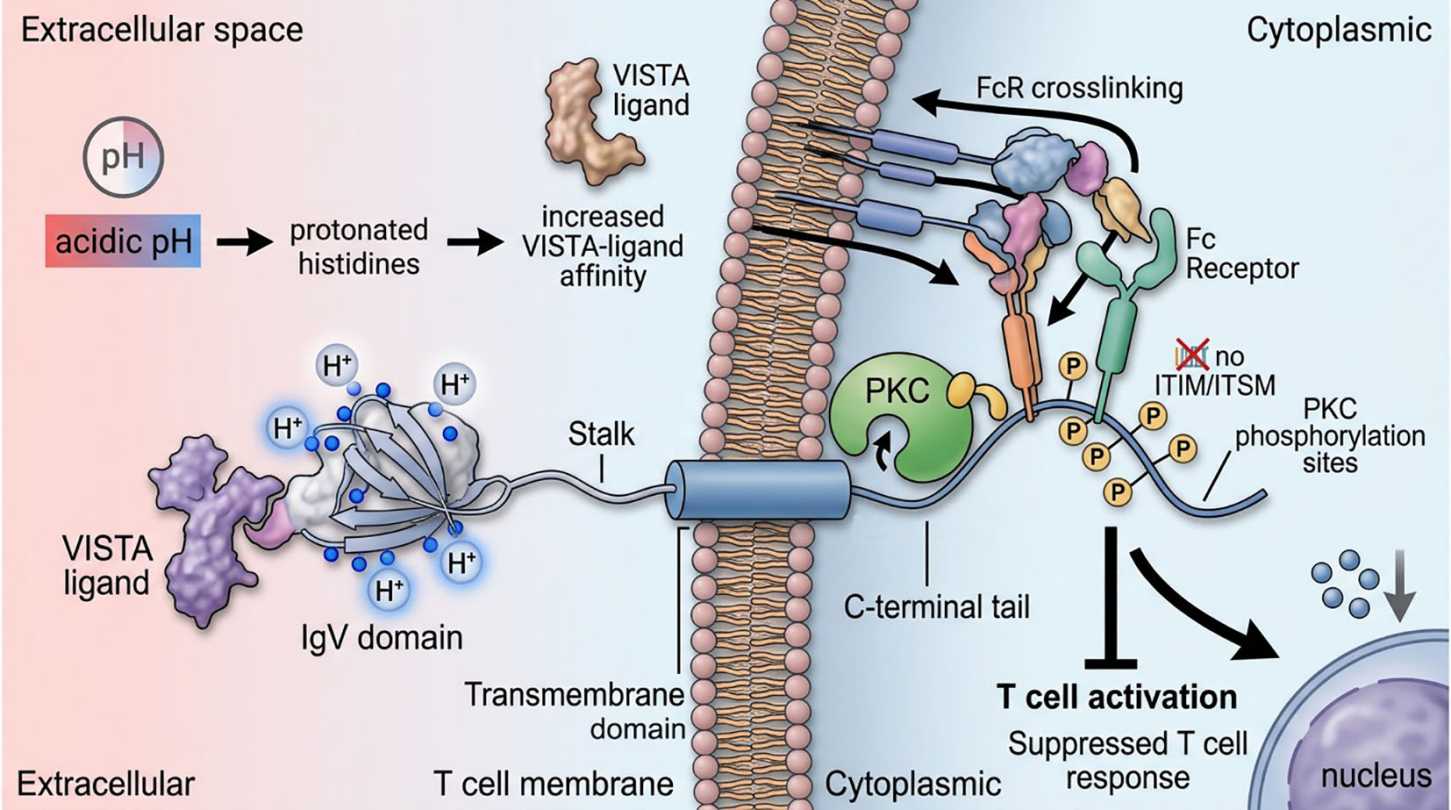
Schematic illustration of VISTA structure, pH-dependent ligand binding, and inhibitory signaling in T cells. VISTA is a type I transmembrane immunoregulatory protein of the B7-CD28 superfamily, expressed on the T cell membrane. The extracellular region consists of an N-terminal IgV-like domain and a short stalk segment. The IgV domain is enriched in histidine residues, which act as a pH-sensitive molecular switch: under acidic pH conditions (e.g., in the TME or inflamed tissues), protonation of these histidines significantly increases VISTA’s binding affinity for its cognate ligands. VISTA also contains a single transmembrane helix and a highly conserved cytoplasmic C-terminal tail. Unlike classical inhibitory checkpoints (e.g., PD-1), the VISTA cytoplasmic tail lacks canonical immunoreceptor tyrosine-based inhibitory motifs (ITIMs) or immunoreceptor tyrosine-based switch motifs (ITSMs), but instead harbors multiple conserved protein kinase C (PKC) phosphorylation sites. VISTA-mediated T cell suppression can be triggered by Fc receptor (FcR)-dependent receptor crosslinking and PKC-mediated phosphorylation of its cytoplasmic tail, which initiates non-canonical intracellular signaling that ultimately inhibits T cell activation and effector function.

VISTA is a type I transmembrane protein, consisting of four core structural domains: an extracellular N-terminal IgV-like domain, a short extracellular stalk region, a single transmembrane helix domain, and a highly conserved cytoplasmic C-terminal tail (28). High-resolution structural analyses have revealed that the IgV domain of VISTA possesses a unique protein fold topology and surface charge distribution that are distinct from those of canonical B7 family members (29). This domain contains multiple conserved disulfide bonds, which confer significant structural rigidity and form a non-canonical ligand-binding interface. Notably, the extracellular IgV domain is enriched in histidine residues, which confer a pH-sensitive molecular switch (30, 31). Protonation of these histidine residues under acidic conditions markedly enhances the binding affinity of VISTA to its cognate ligands. This unique property indicates that VISTA-mediated immunosuppressive signaling may be preferentially augmented in specific tissue microenvironments with low pH, such as inflamed tissues during sepsis or the tumor microenvironment (TME). In contrast to the divergent extracellular domain, the cytoplasmic tail of VISTA is highly conserved across mammalian species, with over 90% sequence homology between human and murine orthologs. Critically, unlike classical inhibitory immune checkpoints such as PD-1, the cytoplasmic tail of VISTA does not contain canonical immunoreceptor tyrosine-based inhibitory motifs (ITIMs) or immunoreceptor tyrosine-based switch motifs (ITSMs), it harbors proline-rich SH3-binding motifs, suggesting signaling through adaptor proteins. Downstream effects of VISTA–ligand engagement are predominantly inhibitory, suppressing T cell proliferation, reducing pro-inflammatory cytokine (IFN-γ, IL-2, IL-17) and chemokine (CCL3, CCL5, CXCL11) production, and limiting immune cell infiltration into the TME (32). Notably, MMP-13 binding to VISTA on osteoclasts induces bone resorption, while also inhibiting T cell activity (33). Instead, it harbors multiple conserved putative protein kinase C (PKC) phosphorylation sites and non-canonical signaling motifs (34). These unique structural and sequence features indicate that the intracellular signal transduction of VISTA is mechanistically distinct from the canonical negative feedback signaling pathways of PD-1. Instead, VISTA-mediated signaling is likely dependent on the assembly of atypical membrane-proximal signaling complexes and Fc receptor-dependent receptor crosslinking (26, 28, 35, 36) (**Figure 1**).

### 2.2 Expression pattern and role in tumor immunity

**Figure 2:**
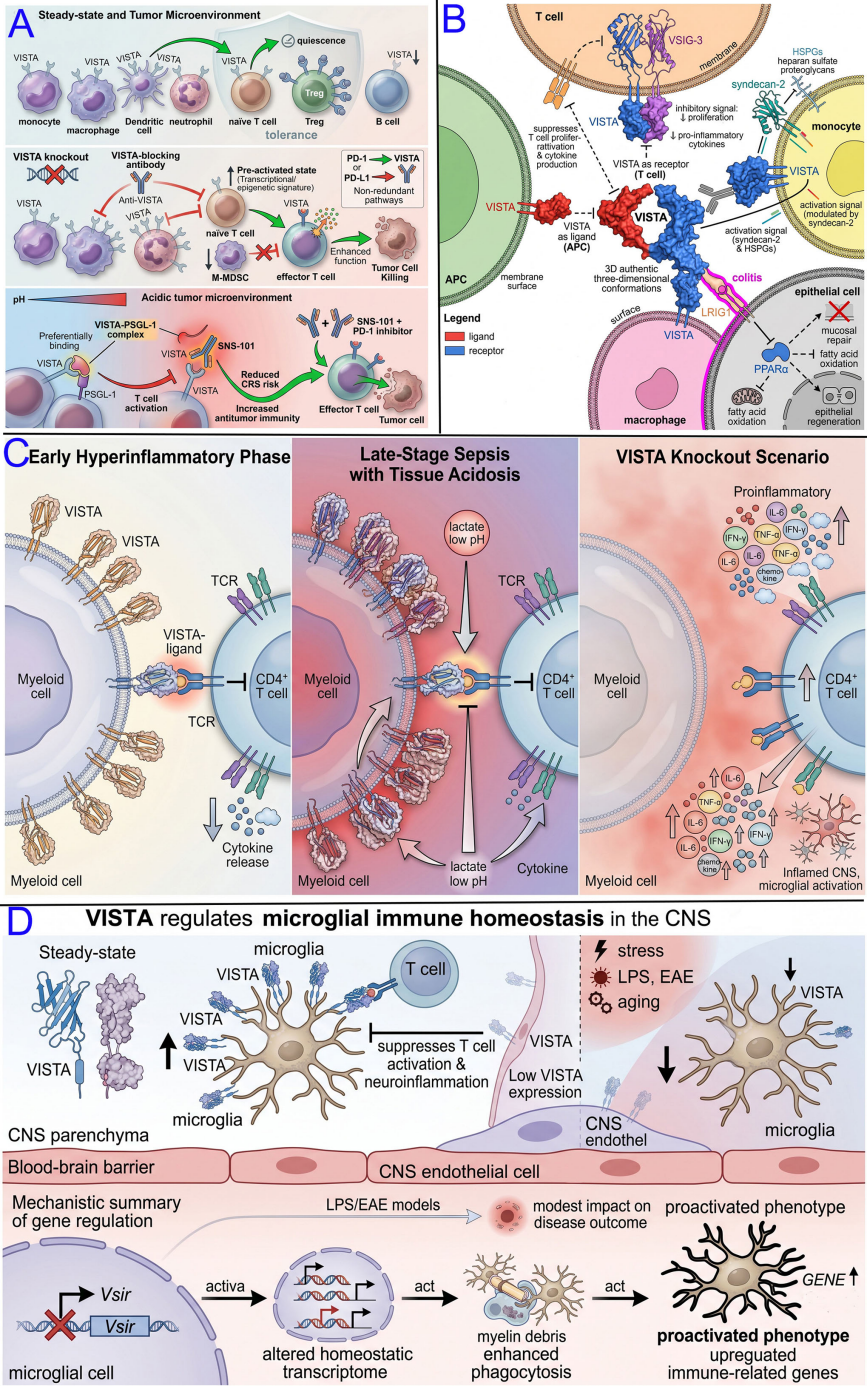
The multifaceted roles of VISTA in immunity, inflammation, and tissue homeostasis. **(A)** VISTA expression and function in steady-state immunity and the tumor microenvironment. VISTA is constitutively expressed across myeloid lineages (monocytes, macrophages, dendritic cells, neutrophils) and naïve/regulatory T cells (Tregs), with low expression on B cells. Under homeostatic conditions, it maintains naïve T cell quiescence and peripheral tolerance. VISTA deficiency or blockade disrupts T cell quiescence, inducing a pre-activated transcriptional/epigenetic state, reducing monocytic myeloid-derived suppressor cells (M-MDSCs), and enhancing effector T cell-mediated tumor killing. VISTA preferentially binds PSGL-1 in the acidic TME. The pH-selective anti-VISTA antibody SNS-101, designed to target this interaction, reduces cytokine release syndrome (CRS) risk and augments antitumor immunity when combined with PD-1 inhibitors, currently in phase I trials. **(B)** VISTA as a bidirectional signaling hub with a multi-ligand interaction network. As a ligand on antigen-presenting cells (APCs), VISTA binds the receptor VSIG-3 on T cells to suppress proliferation and proinflammatory cytokine production. As a receptor on T cells, VISTA delivers cell-intrinsic inhibitory signals. On human monocytes, VISTA can also act as an activating receptor, with its signaling modulated by syndecan-2 and heparan sulfate proteoglycans (HSPGs). In colitis, macrophage-derived VISTA interacts with LRIG1 on epithelial cells, inhibiting PPARα signaling and impairing fatty acid oxidation, thereby disrupting mucosal regeneration and barrier repair. **(C)** Context-dependent VISTA function in sepsis and inflammatory disorders. In early hyperinflammatory sepsis, high VISTA expression on myeloid cells limits excessive T cell activation and cytokine release, conferring tissue protection. In late-stage sepsis with tissue acidosis (low pH from lactate accumulation), the acidic environment amplifies VISTA-mediated immunosuppressive signaling, creating a feedforward loop that exacerbates immune paralysis. VISTA knockout leads to spontaneous proinflammatory T cell activation, elevated cytokines, and exacerbated inflammation, highlighting its role in calibrating inflammatory thresholds and promoting tissue tolerance. **(D)** VISTA regulation of microglial immune homeostasis in the central nervous system (CNS). VISTA is predominantly expressed by microglia in the CNS. Under steady-state conditions, microglial VISTA suppresses T cell activation and neuroinflammation to maintain immune homeostasis. Stressors including LPS, EAE, and aging downregulate VISTA expression, promoting a proactivated microglial phenotype. Conditional deletion of the VISTA-encoding gene Vsir remodels the microglial transcriptome, enhances myelin phagocytosis, and upregulates immune-related genes, though its impact on disease progression in LPS/EAE models is modest.

VISTA exhibits a highly cell-type-specific expression pattern that distinguishes it from other canonical immune checkpoints (25, 26, 37). It is constitutively and highly expressed across myeloid lineages, including monocytes, macrophages, dendritic cells, and neutrophils (38). VISTA is also stably expressed on naïve T cells and regulatory T cells (Tregs), whereas its expression on B cells is relatively low. In contrast to CTLA-4 and PD-1, whose expression is dynamically upregulated following T cell activation, VISTA is readily detectable on myeloid cells and specific T cell subsets under steady-state conditions. Cumulative evidence indicates that endogenous VISTA on naïve T cells serves as an early negative checkpoint, critical for maintaining cellular quiescence and peripheral immune tolerance (25, 39). Concordantly, VISTA deficiency diminishes the quiescent naïve T cell compartment and induces a transcriptional and epigenetic signature consistent with a pre-activated state. Clinical and preclinical observations further support VISTA as a functionally nonredundant checkpoint pathway, mechanistically distinct from the PD-1/PD-L1 axis (27, 40, 41). Genetic or antibody-mediated VISTA blockade reduces the frequency of monocytic myeloid-derived suppressor cells (M-MDSCs) and enhances effector T cell function, thereby suppressing tumor growth across multiple preclinical models (42). Leveraging the preferential binding of VISTA to PSGL-1 in acidic microenvironments, a pH-selective anti-VISTA antibody (SNS-101) has been engineered. This innovative strategy is designed to minimize systemic peripheral clearance, reduce the risk of cytokine release syndrome (CRS), and augment antitumor efficacy when combined with PD-1 inhibitors. SNS-101 is currently undergoing phase I clinical evaluation (43). Nevertheless, given VISTA’s dual role in facilitating tumor immune escape and sustaining normal tissue immune homeostasis, the successful clinical translation of VISTA-targeted therapies will necessitate the development of refined biomarker-driven strategies to guide patient stratification and enhance safety monitoring (37, 40) (**Figure 2A**).

### 2.3 Dual functionality and interaction network of VISTA

VISTA occupies a unique and multifaceted position within the immune checkpoint signaling network, distinguished primarily by its capacity for bidirectional signaling as both a ligand and a receptor (27, 44, 45). Early foundational studies characterized VISTA as an inhibitory ligand on antigen-presenting cells (APCs), where it engaged an initially unidentified receptor on T cells to suppress proliferation and cytokine production (25, 26). Concurrently, VISTA was shown to function as a coinhibitory receptor on T cells themselves, directly restricting CD4^+^ T cell effector responses. Consistent with this dual inhibitory role, agonistic anti-VISTA antibodies exhibit profound immunosuppressive activity in preclinical models of inflammation (46).

VISTA interacts with multiple ligands, the most well-characterized being VSIG-3 and PSGL-1, along with less-confirmed ones such as Gal-9, VSIG-8, MMP-13, Sdc2, and LRIG1 (32). More recent mechanistic investigations have refined this model, emphasizing the critical importance of contextual dependence, particularly pH. Under acidic conditions (pH ~6.0), VISTA acts as a selective ligand for P-selectin glycoprotein ligand-1 (PSGL-1), potently suppressing antitumor T cell activity (30); this interaction is markedly attenuated at neutral pH. This pH-dependent binding profile aligns precisely with the acidic microenvironments characteristic of solid tumors and acutely inflamed tissues, biological contexts highly relevant to sepsis pathophysiology. While the precise downstream signaling pathways following VISTA-PSGL-1 ligation remain uncharacterized, studies in mice show that PSGL-1 engagement can inhibit T cell receptor (TCR) signaling, reducing phospho-ERK, AKT, and STAT5, thereby promoting T cell exhaustion (47). Furthermore, when functioning as a receptor, VISTA transduces cell-intrinsic inhibitory signals upon ligation. V-set and immunoglobulin domain-containing 3 (VSIG-3) has been identified as a cognate ligand for VISTA, at physiological pH (7.4), which delivers inhibitory signals into T cells, thereby suppressing human T cell proliferation and reducing the production of multiple proinflammatory cytokines, such as IL-2, IFN-γ, and IL-17 (48) (**Figure 2B**). The downstream signaling pathways for both VISTA and its ligands PSGL-1 and VSIG3 are not fully understood and represent a key outstanding question in the field. The acidic TME potentiates VISTA’s immunoinhibitory activity via PSGL-1 binding, contributing to tumor immune evasion (35). Consequently, VISTA is a promising target for cancer immunotherapy, with strategies including blocking antibodies to disrupt its inhibitory signals. Furthermore, modulating the intratumoral pH to neutralize the TME could be a novel approach to inhibit the VISTA-PSGL-1 axis and enhance anti-tumor immunity (27).

Beyond T cell regulation, VISTA exerts context-dependent effects on myeloid cells. Specifically, VISTA can function as an activating receptor on human monocytes following Fc receptor-mediated cross-linking, with its binding affinity and signaling capacity modulated by syndecan-2 and heparan sulfate proteoglycans (49, 50) (**Figure 2B**). Collectively, these findings establish VISTA as the hub of a complex, multi-ligand, multi-cell-type interaction network governed by its bidirectional signaling potential. Notably, VISTA’s biological influence extends beyond canonical immune inhibition to encompass tissue repair and metabolic regulation. In the setting of colitis, macrophage-derived VISTA has been demonstrated to interact with leucine-rich repeats and immunoglobulin-like domains 1 (LRIG1), a molecule traditionally linked to epithelial biology and stem cell homeostasis (51). This interaction suppresses peroxisome proliferator-activated receptor α (PPARα) signaling, disrupting fatty acid oxidation and epithelial regeneration, which ultimately impairs mucosal barrier repair. This newly identified immune–metabolic–tissue repair axis suggests that VISTA may directly contribute to mucosal injury and dysregulated regeneration. Given the central role of intestinal barrier dysfunction in sepsis pathogenesis, this non-canonical pathway highlights a potentially critical and underappreciated mechanism by which VISTA could influence organ injury during sepsis. A recently identified ligand with restricted expression in stratified epithelia and some immune cells. VSIG-8–VISTA interactions moderately inhibit T cell activation and contribute to tissue tolerance in mucosal and skin barriers. Its role in cancer remains understudied, but it may modulate local immune responses in epithelial malignancies (32).

### 2.4 Context-dependent functions in inflammatory diseases and sepsis

VISTA exerts context-dependent, dual regulatory effects across a spectrum of inflammatory disorders, with its functional role being highly contingent on the stage and severity of disease. During the early hyperinflammatory phase of sepsis or in the setting of autoimmunity, VISTA, whose high constitutive expression on myeloid cells is a defining feature, may confer tissue protection by limiting excessive T cell activation and mitigating the cytokine storm (26, 36). Conversely, in late-stage sepsis characterized by immune paralysis, sustained VISTA overexpression may exacerbate immunosuppression. Furthermore, the local tissue acidosis induced by hypoperfusion and lactate accumulation in sepsis could amplify VISTA-mediated signaling via its pH-sensitive binding mechanism, thereby creating a feedforward loop that intensifies immunosuppression (30, 52). Genetic ablation of VISTA results in age-associated spontaneous T cell activation, accompanied by elevated expression of proinflammatory cytokines and chemokines. In genetically susceptible backgrounds, VISTA deficiency markedly increases both the incidence and severity of experimental autoimmune encephalomyelitis (EAE) (53). As a coinhibitory receptor, VISTA appears to preferentially constrain CD4^+^ T cell-driven immunopathology (46), underscoring its role in calibrating inflammatory thresholds and promoting tissue tolerance (**Figure 2C**).

### 2.5 Expression and function in the central nervous system

VISTA exhibits a distinctive expression and functional profile within the central nervous system (CNS). It is predominantly expressed by microglia, with relatively low expression detected on CNS endothelial cells (54). Under steady-state conditions, microglial VISTA suppresses excessive T cell activation and neuroinflammation, thereby sustaining CNS immune homeostasis (55, 56). Borggrewe et al. demonstrated that microglial VISTA expression is downregulated in response to various stressors, including lipopolysaccharide (LPS) stimulation, EAE induction, and aging (54). In clinical samples, VISTA expression displays disease-associated alterations in lesions from patients with multiple sclerosis and in the brains of patients with Alzheimer’s disease. Moreover, conditional deletion of *Vsir* (the gene encoding VISTA) reshapes the microglial homeostatic transcriptome and modulates myelin phagocytosis, thereby influencing intracerebral immune balance (55, 57). Although microglia-specific VISTA deletion in *Cx3cr1*-lineage cells promotes a proactivated microglial phenotype and upregulates immune-related gene expression, its impact on overall disease progression in LPS and EAE models appears to be modest (58). Collectively, these findings position VISTA as a key regulator of microglial homeostasis. While reduced VISTA expression likely facilitates the initiation and amplification of neuroinflammatory responses, the precise context-dependent roles of VISTA across distinct neurological diseases and CNS regions remain incompletely defined and warrant further investigation (**Figure 2D**).

## 3 VISTA: a bidirectional immune checkpoint in sepsis pathophysiology and organ injury

### 3.1 The role of VISTA in sepsis pathophysiology

The hyperinflammatory phase of sepsis is pathologically defined by explosive, uncontrolled activation of the innate immune system: pathogen-associated molecular patterns (PAMPs) from invading pathogens and damage-associated molecular patterns (DAMPs) released from injured host cells engage pattern recognition receptors, most notably Toll-like receptors (TLRs), on innate immune cells (4, 7, 58). This signaling cascade drives robust, dysregulated production of a broad panel of proinflammatory mediators by circulating monocytes, tissue-resident macrophages, and neutrophils. In parallel, this hyperinflammatory state triggers widespread activation of the complement system, coagulation cascade, and vascular endothelium, events that collectively culminate in microcirculatory dysfunction, tissue injury, and progressive multiple organ dysfunction syndrome.

However, accumulating clinical observations and autopsy studies have established that persistent immunosuppression (also termed immune paralysis) is the predominant driver of late sepsis-related morbidity and mortality (59). This paradigm shift in our understanding of sepsis immunobiology has redirected research focus from non-specific global anti-inflammatory strategies to a more nuanced, stage-specific understanding of dynamic immune remodeling. It is now widely recognized that inappropriate or premature immune interventions may exacerbate tissue injury, worsen immune dysregulation, and increase mortality risk. In this context, immune checkpoint molecules have emerged as critical bidirectional regulators of sepsis-associated immune dysfunction, and as promising targets for precision immunomodulation. These pathways exert context-dependent effects: they may constrain excessive inflammatory activation and protect against bystander tissue injury in the early hyperinflammatory phase of sepsis, while sustained upregulation of these checkpoints may drive and maintain pathological immunosuppression (16, 17, 60). Furthermore, the expression profiles of immune checkpoint molecules can serve as actionable biomarkers of host immune status, enabling individualized patient stratification, prognostic assessment, and guidance of timing-specific immunotherapeutic interventions. Among these checkpoints, VISTA stands out due to its unique constitutive expression on myeloid cells, bidirectional receptor-ligand signaling capacity, and pH-sensitive functional properties, which confer it with a distinctive and non-redundant role in modulating the biphasic immune response in sepsis.

VISTA is an emerging non-canonical negative immune checkpoint that represents a promising target for advancing our mechanistic understanding of sepsis pathophysiology and guiding the development of precision immunomodulatory strategies. However, the dynamic expression profile of VISTA across the sepsis disease course remains incompletely reconciled across preclinical studies, with available evidence demonstrating marked spatiotemporal heterogeneity shaped by pathogen burden, tissue microenvironment, and the therapeutic time window of assessment. In a cecal ligation and puncture (CLP) model of polymicrobial septic peritonitis, Tao et al. reported constitutively high VISTA expression on T cells and macrophages, with no significant changes in expression detected within 24–72 h after sepsis induction (22). Notably, despite the stable expression profile, administration of the high-affinity anti-VISTA antibody MH5A attenuated systemic inflammation and improved 7-day survival in septic mice. In contrast, Hu et al. employed a two-hit hemorrhagic shock plus CLP model of indirect acute respiratory distress syndrome (iARDS), and found that VISTA was markedly upregulated on circulating blood monocytes, alveolar macrophages, circulating and lung-infiltrating neutrophils, as well as lung epithelial and endothelial cells (61). This upregulation of VISTA paralleled elevated levels of inflammatory and chemotactic mediators and the severity of acute lung injury, leading the authors to propose a protective, homeostatic checkpoint role for VISTA in this model (62). Additionally, Gray et al. demonstrated that VISTA expression was significantly increased on CD4^+^ regulatory T cells (Tregs) following septic challenge in wild-type mice (23). Genetic VISTA deficiency impaired the early maintenance and expansion of CD4^+^ Tregs, which was associated with worse acute disease recovery and higher mortality. These seemingly discrepant observations are likely attributable to key differences in preclinical model systems, including the severity of the septic insult, timing of sample collection, anatomical compartment of sampling, and the epitope specificity of the anti-VISTA antibody clones used. However, systematic head-to-head comparisons controlling for these variables are currently lacking. Future studies should prioritize standardized, multi-center preclinical trials that prospectively evaluate VISTA expression dynamics across defined sepsis stages, tissue compartments, and model severities to resolve these discrepancies and establish a unified framework. Importantly, these findings highlight that the expression and functional activity of VISTA are dynamically regulated in a cell-type-specific, tissue-niche-specific, and disease-stage-dependent manner during sepsis. The proposed biphasic model is a synthesis of findings from distinct preclinical models (e.g., CLP, LPS, iARDS) with varying severities and timelines, and that no single study has yet demonstrated these opposing effects within a controlled, time-series experiment.

Emerging evidence indicates that VISTA is expressed on various epithelial and endothelial cells, including those lining the lungs, gut, and the blood-brain barrier (63). Under steady-state conditions, this expression may contribute to barrier integrity through the following mechanisms. VISTA signaling may help maintain tight junctions between epithelial and endothelial cells by influencing cytoskeletal rearrangement and the expression of tight junction proteins. This prevents the non-specific leakage of pathogens or macromolecules (64). Under homeostatic conditions, VISTA on barrier cells can interact with ligands (e.g., PSGL-1) on local tissue-resident immune cells (e.g., macrophages, dendritic cells), transmitting inhibitory signals. This suppresses excessive responses by these immune cells to low-level, harmless environmental antigens or commensal microbes. This helps maintain a state of “immune silence,” preventing unnecessary inflammatory activation that could damage barrier structures (27).

VISTA’s regulatory role in immune cell migration under steady-state conditions is another key aspect of its non-immune cell function. VISTA on vascular endothelial cells may fine-tune the migration of leukocytes (e.g., neutrophils, monocytes) from the blood into tissues by influencing the local expression or presentation of chemokines (e.g., CXCL8, CCL2). Under steady-state conditions, this regulation likely helps maintain a population of “sentinel” cells within tissues while preventing excessive, non-specific immune cell infiltration (64). Circulating immune cells, provided they express VISTA ligands (e.g., PSGL-1), may undergo transient interactions with VISTA on vascular endothelial cells. This interaction could serve as a “docking” or “checkpoint” signal, regulating immune cell rolling, adhesion, and eventual transmigration across the endothelial barrier. Under homeostatic conditions, this mechanism ensures that only appropriately activated or functionally specific immune cells gain access to the tissue parenchyma, thereby maintaining immune homeostasis within the tissue microenvironment (65). On endothelial cells of the blood-brain barrier, VISTA expression may contribute to the CNS’s immune-privileged status. By interacting with ligands on circulating immune cells, VISTA can actively inhibit their transmigration, thereby protecting neural tissue from peripheral immune attack (57). On intestinal epithelial cells, VISTA expression is crucial for managing coexistence with the vast commensal microbial community. It helps establish immune tolerance toward the commensal flora, preventing inflammatory reactions against harmless antigens, thereby maintaining the gut’s barrier function and a homeostatic environment for nutrient absorption (64).

### 3.2 Regulation of myeloid cell function by VISTA

**Figure 3:**
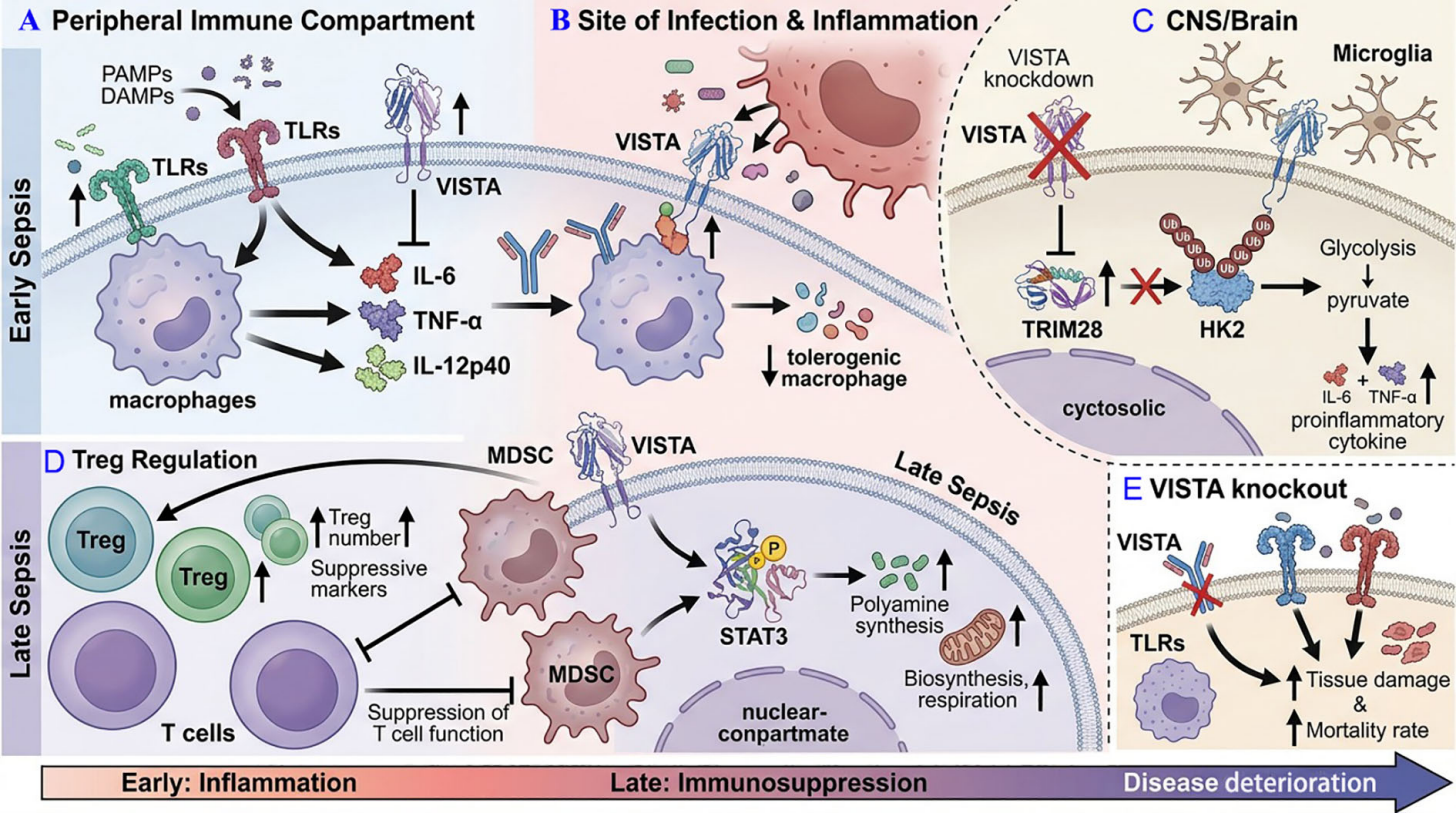
Stage-specific and cell-type-specific functions of VISTA in sepsis pathophysiology and organ injury. **(A)** VISTA-mediated regulation of the early hyperinflammatory phase in the peripheral immune compartment. During early sepsis, pathogen-associated molecular patterns (PAMPs) and damage-associated molecular patterns (DAMPs) activate Toll-like receptors (TLRs) on macrophages, driving robust production of proinflammatory cytokines including IL-6, TNF-α, and IL-12p40. VISTA is constitutively expressed on myeloid cells and acts as a critical rheostat to constrain excessive TLR-driven inflammatory activation, limiting pathological cytokine storm and mitigating bystander tissue injury. **(B)** VISTA-dependent tolerogenic macrophage polarization at sites of infection and inflammation. At local inflammatory foci, VISTA engagement induces a tolerogenic macrophage phenotype, characterized by reduced proinflammatory mediator release and enhanced immune suppressive functions. This VISTA-mediated polarization serves as a homeostatic mechanism to dampen local inflammation and preserve tissue integrity during acute septic challenge. **(C)** VISTA knockout drives severe tissue damage and mortality in late sepsis. Genetic ablation of VISTA abrogates the immune regulatory checkpoint, leading to dysregulated TLR signaling, unchecked inflammatory cytokine production, and severe tissue damage across multiple organs. This unrestrained immune activation culminates in a marked increase in mortality rates, highlighting VISTA’s non-redundant role in maintaining immune homeostasis and survival during late-stage sepsis. **(D)** VISTA regulation of microglial glycolysis and neuroinflammation in sepsis-associated encephalopathy (SAE). In the CNS/brain, VISTA is highly expressed by homeostatic microglia, where it suppresses neuroinflammation by limiting glycolytic metabolism. VISTA knockdown upregulates the E3 ubiquitin ligase TRIM28, which promotes K63-linked ubiquitination and stabilization of hexokinase 2 (HK2), a key glycolytic enzyme. Enhanced HK2-mediated glycolysis amplifies microglial proinflammatory activation, increasing the release of TNF-α, IL-6, and other proinflammatory cytokines and exacerbating SAE pathogenesis. **(E)** VISTA sustains immunosuppression via MDSC expansion and Treg regulation in late sepsis. During late sepsis, VISTA signaling drives the differentiation and functional activation of myeloid-derived suppressor cells (MDSCs). VISTA promotes STAT3 activation and polyamine biosynthesis in MDSCs, supporting mitochondrial respiration and enhancing their immunosuppressive capacity to suppress T cell function. Concurrently, VISTA maintains the number and suppressive marker expression of regulatory T cells (Tregs), creating a robust immune suppressive microenvironment that drives sepsis-induced immune paralysis and secondary infection susceptibility.

The hyperinflammatory cascade and subsequent organ injury in sepsis are predominantly driven by aberrant pattern recognition receptor (PRR) signaling in myeloid lineage cells, including circulating monocytes, tissue-resident macrophages, and neutrophils (4, 8, 59). Given the constitutively high expression of VISTA across myeloid subsets, it is biologically plausible that VISTA functions as a key rheostat to calibrate the activation threshold of TLR and other PRR-triggered inflammatory pathways (26, 32, 35). Through this regulatory activity, VISTA can broadly modulate myeloid cell effector functions, including cytokine and chemokine production, chemotaxis, phagocytosis, and migratory capacity. Cumulative preclinical evidence predominantly supports a model in which VISTA restrains excessive innate immune activation, thereby protecting against dysregulated systemic inflammatory responses during the early phase of sepsis (35). ElTanbouly et al. demonstrated that agonistic anti-VISTA antibodies induced transcriptional and epigenetic reprogramming in macrophages, which downregulated the expression of proinflammatory cytokines including IL-6, TNF, and IL-12p40, and stabilized an anti-inflammatory macrophage phenotype (66). This reprogramming increased host tolerance to lipopolysaccharide LPS challenge and reduced LPS-induced lethality in murine models. Consistent with these observations, Vivian et al. found that LPS stimulation rapidly upregulated surface expression of VISTA on murine macrophages and neutrophils (**Figure 3A**). Administration of a high-affinity VISTA ligand, VISTA.COMP, significantly attenuated LPS-induced production of TNF, IL-6, and IL-12p40, and reversed the proinflammatory phenotype of both myeloid cell subsets (67). Collectively, these results indicate that VISTA activation limits inflammatory amplification and promotes an anti-inflammatory phenotype in human monocytes under hyperinflammatory conditions (68). *In vitro* studies further corroborate these findings: the high-affinity anti-VISTA antibody MH5A, when ligated to the VISTA receptor, effectively suppressed LPS-induced proinflammatory cytokine production by primary macrophages (22). Conversely, VISTA may enhance immunosuppressive function in specific myeloid subsets under certain disease contexts. Lee and colleagues reported that VSIG4^+^ macrophages expanded in the peritoneal cavity after CLP, and produced higher levels of nitric oxide in response to VISTA stimulation (69). These VSIG4^+^ macrophages also exhibited enhanced suppressive capacity against T cell proliferation. Thus, in terminally differentiated immunosuppressive myeloid populations, VISTA signaling may further reinforce their tolerogenic and anti-inflammatory functions (**Figure 3B**).

### 3.3 VISTA in sepsis-associated encephalopathy and peripheral organ injury

Sepsis-associated encephalopathy (SAE) is a frequent and devastating complication of sepsis, with a complex, multifactorial pathophysiology. Clinically, SAE encompasses a broad spectrum of neurological manifestations, ranging from acute delirium to deep coma, and is increasingly recognized as a critical determinant of both short-term mortality and long-term neurocognitive outcomes in septic patients (70–74). The core pathophysiological mechanisms of SAE center on microglial activation, neuroinflammation, and immunometabolic remodeling, which are driven by systemic inflammation and immune dysregulation, forming self-reinforcing pathological feedback loops (75–79). Preclinical studies have demonstrated that microglia constitutively express high levels of VISTA, and that proinflammatory stimulation downregulates microglial VISTA expression, providing biological plausibility for a mechanistic role of VISTA in SAE pathogenesis (57, 68). Xu and colleagues investigated the mechanistic role of VISTA in negatively regulating microglial glycolysis and neuroinflammation in a murine CLP model of SAE (80). They found that sepsis induced a significant downregulation of VISTA expression in hippocampal microglia, which was accompanied by increased expression of the microglial activation marker CD68 and enrichment of proinflammatory transcriptional programs, including TNF and NF-κB signaling pathways. *In vitro* studies, VISTA knockdown in BV2 microglial cells upregulated the expression of the E3 ubiquitin ligase TRIM28, which in turn promoted K63-linked ubiquitination of the key glycolytic enzyme hexokinase 2 (HK2). This post-translational modification reduced proteasomal degradation of HK2 and enhanced microglial glycolysis, ultimately amplifying LPS-induced release of proinflammatory mediators including TNF-α, IL-6, and CCL3. These results suggest that VISTA may protect against SAE, potentially by limiting microglial metabolic reprogramming and proinflammatory activation. This protective role is supported by *in vitro* experiments showing that VISTA knockdown enhances glycolysis and cytokine release, via a TRIM28-associated ubiquitination pathway that destabilizes HK2, although further *in vivo* loss- and gain-of-function studies are needed to establish causality (**Figure 3C**).

### 3.4 VISTA in the late immunosuppressive phase and myeloid-derived suppressor cells

In the immunosuppressive phase of sepsis, expansion of MDSCs is a well-documented pathological event, which is closely associated with T cell functional suppression and increased susceptibility to secondary nosocomial infections (9, 60, 81). While the role of the VISTA-MDSC axis has been predominantly investigated in the TME, these mechanistic insights are highly relevant to sepsis-induced myeloid remodeling. Zhang and colleagues reported that VISTA signaling sustains STAT3 activation and promotes polyamine biosynthesis in myeloid cells (82). These metabolic and transcriptional changes drive MDSC differentiation and support mitochondrial respiration, thereby enhancing the immunosuppressive capacity of MDSCs. If analogous pathways are operational during sepsis-induced myeloid remodeling, VISTA may exert dual, stage-specific effects: limiting early inflammatory amplification during the hyperinflammatory phase, while deepening immune paralysis in the late disease stage by promoting the expansion and functional activation of suppressive myeloid subsets. This dual-effect model also helps reconcile seemingly discrepant findings regarding the efficacy of VISTA blockade in preclinical sepsis models. Specifically, VISTA blockade may attenuate T cell apoptosis and improve disease outcomes by dampening the generation and suppressive function of MDSCs, thereby indirectly restoring effector T cell activity (**Figure 3D**).

### 3.5 Modulation of lymphocyte responses by VISTA

Progressive lymphocyte apoptosis and profound T cell functional exhaustion are core pathological mechanisms underlying sepsis-induced immunosuppression (9, 18). Studies investigating the role of VISTA in lymphocyte regulation during sepsis have revealed substantial functional complexity, with context-dependent effects on distinct lymphocyte subsets. Tao and colleagues demonstrated that treatment with the anti-VISTA antibody MH5A significantly reduced apoptosis of CD3^+^ T cells in the spleen and thymus of CLP septic mice (22). This intervention improved overall survival and enhanced bacterial clearance in the peritoneal cavity and bloodstream. These findings suggest that, under specific conditions, blockade of VISTA signaling may relieve inhibitory pathways that drive T cell apoptosis and exhaustion, thereby restoring T cell survival and effector function.

In contrast, while expansion of Tregs is widely recognized as a hallmark of sepsis-induced immunosuppression, Tregs also play a non-redundant, tissue-protective role during the acute hyperinflammatory phase by limiting bystander tissue injury. Gray and colleagues demonstrated that VISTA plays a critical, non-redundant role in maintaining Treg numbers and supporting their suppressive function during acute sepsis (23). In VISTA-knockout (VISTA^−/−^) mice, the loss of Tregs was accompanied by dysregulated control of systemic inflammation and significantly increased mortality. Importantly, adoptive transfer of VISTA-sufficient Tregs rescued the survival of VISTA^−/−^ septic mice to levels comparable to those of wild-type controls (**Figure 3E**). These results indicate that VISTA-mediated maintenance of Treg-dependent immune braking is protective during the acute inflammatory phase of sepsis. More broadly, these findings challenge the paradigm of VISTA as a purely suppressive checkpoint, and instead support a model in which VISTA functions to establish a controllable immune steady state during intense inflammatory stress. Gray et al. further identified an additional regulatory axis of VISTA in innate-like lymphocytes, finding that VISTA deficiency led to abnormal accumulation of intestinal CD69low γδ T cells after CLP, a subset that did not exhibit tissue-protective activity in their model (24). Adoptive transfer of Jurkatfoxp3 Tregs reduced the expansion of this pathogenic intestinal γδ T cell subset and shifted the remaining population toward a protective CD69high phenotype. These findings reveal an additional mechanism whereby VISTA shapes the repertoire and functional phenotype of the intestinal γδ T cell compartment during sepsis.

The mechanistic models presented are primarily derived from preclinical work, and the translation of these findings into human sepsis cohorts remains a critical unmet need. We have explicitly noted that without robust clinical validation, the stage-specific model remains a hypothesis requiring direct testing in patient samples.

## 4 VISTA as a context-dependent therapeutic target in sepsis: from preclinical evidence to precision immunotherapy strategies

### 4.1 Current clinical landscape and the rationale for VISTA-targeted therapy in sepsis

Current clinical management of sepsis remains predominantly supportive, with core priorities including early and effective antimicrobial therapy, goal-directed fluid resuscitation, and organ function support. However, these standard-of-care measures do not directly target the dysregulated host immune cascade triggered by PAMPs and DAMPs (60). Consequently, progressive organ injury and a high risk of secondary nosocomial infections persist as major drivers of sepsis-related morbidity and mortality. There is therefore an urgent unmet need for mechanism-based immunotherapies that can simultaneously attenuate excessive inflammatory responses, mitigate end-organ damage, and improve long-term clinical outcomes.

**Figure 4:**
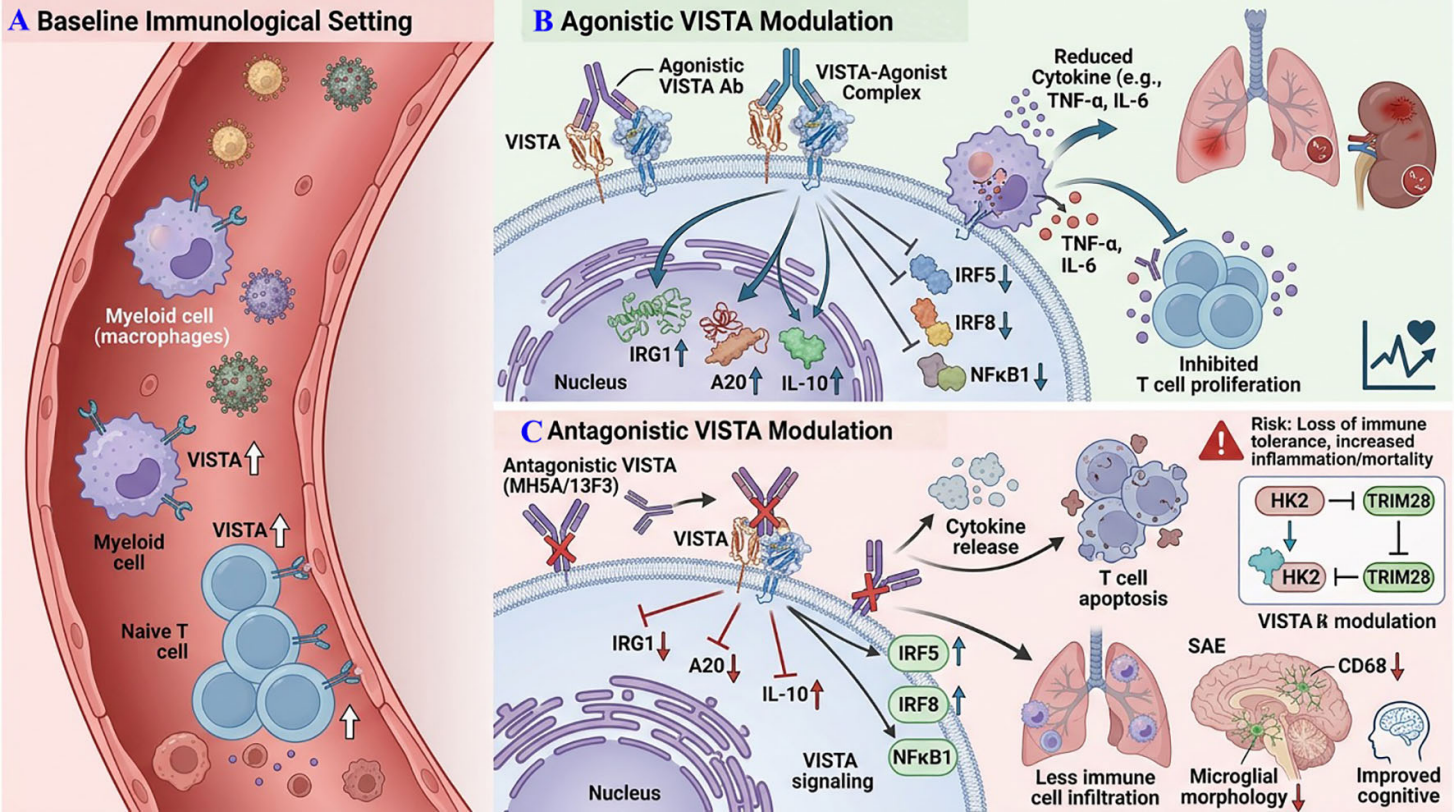
Baseline immune status and bidirectional therapeutic modulation of VISTA in sepsis. **(A)** Baseline immunological setting of VISTA in sepsis. Under steady-state conditions during sepsis, VISTA is constitutively and highly expressed on myeloid cells (including macrophages) and naïve T cells. This cell-type-specific VISTA expression establishes a critical immunological “brake” that calibrates innate and adaptive immune activation thresholds, maintaining peripheral immune homeostasis and preventing aberrant inflammatory responses. This baseline expression profile shapes the functional context for VISTA-targeted therapeutic intervention in septic pathophysiology. **(B)** Agonistic VISTA modulation mitigates hyperinflammation and end-organ injury. Therapeutic activation of VISTA via agonistic antibodies or VISTA-agonist complexes initiates intracellular signaling that upregulates immunoregulatory mediators (e.g., IRG1, A20, IL-10) while suppressing key proinflammatory transcription factors (e.g., IRF5, IRF8, NFκB1). This signaling cascade reduces the production of proinflammatory cytokines (e.g., TNF-α, IL-6) and inhibits pathogenic T cell proliferation. Consequently, VISTA agonism attenuates systemic inflammation, preserves tissue integrity in vital organs (e.g., lungs, kidneys), and ameliorates sepsis-driven end-organ damage. **(C)** Antagonistic VISTA modulation reverses immunosuppression and modulates neuroinflammation. Targeted blockade of VISTA using high-affinity antagonistic antibodies (e.g., MH5A/13F3) abrogates VISTA-mediated inhibitory signaling, leading to enhanced proinflammatory cytokine release and reversal of T cell apoptosis. This intervention reduces pathological immune cell infiltration into peripheral organs (e.g., lungs) and reshapes microglial morphology (e.g., downregulating CD68 expression), thereby alleviating sepsis-associated encephalopathy (SAE) and improving cognitive function. Mechanistically, VISTA antagonism modulates the HK2-TRIM28 axis to regulate microglial glycolysis, mitigating neuroinflammation and restoring neurological homeostasis.

Among immune checkpoint molecules, VISTA is uniquely positioned as a therapeutic target for sepsis due to its constitutively high expression on myeloid lineage cells and resting T cells (25, 68), enabling it to modulate both the early innate inflammatory amplification and late adaptive immune exhaustion that characterize sepsis. Theoretically, targeted modulation of VISTA can re-establish physiological immune homeostasis and improve clinical outcomes in sepsis. Preclinical studies have corroborated this potential, demonstrating that antibody-mediated VISTA modulation reduces cytokine storm, attenuates T cell apoptosis, and improves survival and end-organ injury in murine sepsis models (22) (**Figure 4A**). Notably, recent advances in immune status-guided precision immunotherapy in selected septic patients have established the feasibility of biomarker-informed interventional trial designs, which can be directly adapted to VISTA-targeted therapeutic strategies (19, 83, 84). Early-phase trials of immune-enhancing agents (recombinant IL-7) and checkpoint inhibitors (anti–PD-L1/anti–PD-1) have established critical precedents for dosing regimens, safety monitoring frameworks, and pharmacodynamic endpoints in sepsis populations (85, 86).

Unlike PD-1 and CTLA-4, which are primarily expressed on activated T cells, VISTA is constitutively and highly expressed on myeloid lineage cells (monocytes, macrophages, neutrophils) and resting T cells, enabling it to modulate both the early innate inflammatory amplification and late adaptive immune exhaustion that characterize sepsis (22, 87). While PD-1/PD-L1 and CTLA-4 are primarily associated with T cell exhaustion in the immunosuppressive phase, VISTA plays a dual role: limiting early hyperinflammation while potentially deepening late immune paralysis (27, 88).

### 4.2 Preclinical evidence for agonistic VISTA modulation strategies

Direct preclinical evidence for VISTA-targeted therapy in sepsis is currently derived predominantly from rodent models (**Table 1**). Given the dual receptor-ligand function of VISTA, therapeutic strategies can be broadly categorized into agonistic and antagonistic approaches, aligned with the stage-specific immune dysregulation in sepsis. ElTanbouly et al. evaluated the immunoprotective effects of VISTA activation in a murine endotoxin shock model (66). They found that the induction of tolerance using a VISTA agonistic antibody in combination with low-dose LPS significantly improved survival after a subsequent lethal high-dose LPS challenge. This protection was associated with enhanced macrophage tolerance. VISTA activation increased the expression of immunosuppressive molecules such as IRG1, A20, and IL-10. At the same time, it suppressed key transcription factors that drive inflammation, including IRF5, IRF8, and NFκB1. Consequently, it reduced the release of inflammatory mediators, such as IL-12p40, IL-6, CXCL2, and TNF-α. These findings indicate that VISTA, as a negative checkpoint regulator, can mitigate lethal inflammation by coordinating tolerogenic and anti-inflammatory pathways. In addition, Prodeus et al. developed VISTA.COMP, a high-affinity VISTA receptor agonist (89). This agent effectively inhibited T-cell proliferation and reduced cytokine production. In mouse models of acute inflammation, VISTA.COMP showed strong immunosuppressive effects and lowered pro-inflammatory cytokines, such as TNF-α and IL-6. Vivian et al. further clarified its actions in myeloid cells, particularly in macrophages and neutrophils (67). After LPS stimulation, VISTA receptor expression increased on macrophages and neutrophils. By binding to the VISTA receptor, VISTA.COMP suppressed LPS-induced inflammation, reduced the production of TNF-α, IL-6, and IL-12, and induced immunoregulatory genes, such as TGF-β and LIF. Together, these studies suggest that VISTA and its agonists have therapeutic potential in sepsis. They may reduce inflammatory responses by modulating immune-cell function and could represent a promising immunotherapeutic strategy for inflammatory diseases, including sepsis (**Figure 4B**).

**Table 1:** Intervention strategies targeting VISTA in preclinical studies of sepsis.

Species	Sex	Age/weight	Targeting strategy	Model	Intervention timing	Outcome	Reference
C57BL/6	Male	~5wks	VISTA gene knockout	CLP	Absent throughout	Mortality increased 25%	(23)
C57BL/6J	Male	8–12 wks	VISTA gene knockout	iARDS	Absent throughout	Survival rate decreased 15% and aggravated lung injury (p<0.01)	(62)
C57BL/6	Both	8–10 wks	VISTA agonistic antibody	LPS	Before LPS challenge	Improve survival following lethal LPS challenge (p<0.001)	(66)
C57BL/6	Female	8–10 wks	VISTA.COMP	LPS	Upon stimulation	Reduce pro-inflammatory cytokine levels (p<.0.01) and alleviate inflammatory response (p<0.05)	(67)
C57BL/6	Male	8–12 wks	MH5A	CLP	Postoperative administration	7−day survival rate improved 50% and reduce bacterial burden (p<0.05)	(22)
C57BL/6	Male	6–8 wks	MH5A	CLP (SAE)	Postoperative administration	Improve cognitive function (p<0.05) and alleviate hippocampal inflammatory injury (p<0.05)	(80)
C57BL/6	Male	8–12 wks	13F3	iARDS	Postoperative administration	Alleviate pulmonary inflammatory infiltration (p<.0.01), but TNF-α was elevated in lung tissue (p<0.05)	(62)

### 4.3 Preclinical evidence for antagonistic VISTA modulation strategies

In addition to agonistic approaches, Tao et al. investigated the high-affinity anti-VISTA antibody MH5A in sepsis (22). In a CLP mouse model, postoperative administration of MH5A significantly improved 7-day survival. It also reduced plasma levels of TNF-α, IL-6, IL-10, and IFN-γ, and decreased bacterial burden in blood and peritoneal fluid. Moreover, MH5A reduced T-cell apoptosis in the spleen and thymus. *In vitro* experiments further showed that MH5A inhibited the secretion of inflammatory cytokines by macrophages after LPS stimulation. These results support a protective role of MH5A in sepsis through modulation of immune-cell responses. Xu and colleagues further assessed VISTA targeting in sepsis-associated encephalopathy (80). In CLP mice, intracerebroventricular administration of MH5A improved the activation morphology of hippocampal microglia and reduced CD68 expression. It also alleviated cognitive impairment. Consistently, expression of key inflammatory genes in the hippocampus, including TNF, IL-6 and CCL3, was downregulated. This study also reported that inhibition of HK2 activity or knockdown of TRIM28 could reverse the enhanced glycolysis and cytokine release caused by VISTA deficiency. These findings suggest that metabolic pathways downstream of VISTA may be actionable targets. Accordingly, VISTA activation or interventions aimed at the HK2 ubiquitination and degradation pathway may offer new therapeutic options for neuroinflammation and cognitive dysfunction associated with sepsis. Overall, VISTA targeting may provide dual benefits by suppressing systemic and neural inflammation, reducing immune-cell apoptosis, and modulating glycolysis-related pathways.

In an iARDS model, Hu et al. evaluated the therapeutic potential of the anti-VISTA monoclonal antibody 13F3 (62). 13F3 antibody treatment resulted in a decrease in VISTA surface expression on monocytes, neutrophils, macrophages, epithelial and endothelial cells. This was accompanied by reduced plasma levels of MCP-1, TNF-α, MIP-2, and IL-10. In lung tissue, 13F3 suppressed IL-6 and MIP-2 expression, whereas TNF-α increased, suggesting tissue-specific immunomodulatory effects. The study also showed that VISTA was highly expressed in peritoneal macrophages and circulating myeloid cells in iARDS mice. In the lung, VISTA was mainly enriched in endothelial and epithelial cells. Loss of VISTA signaling increased pulmonary chemokines, such as KC and MIP-2, which promoted excessive recruitment of neutrophils and monocytes into the lung interstitium. In contrast, 13F3 treatment reduced the chemokine gradient and blocked the pathological migration of inflammatory cells into lung tissue. Based on these observations, VISTA may contribute to disease progression not only by regulating cytokines but also by influencing cell trafficking, barrier function, and cell death programs (**Figure 4C**).

### 4.4 Theoretical challenges and context-dependent nature of VISTA intervention

Importantly, the idea of simply blocking VISTA to release immune potential during the immunosuppressive phase of sepsis remains largely theoretical. Robust experimental evidence is still limited. In fact, anti-VISTA antibodies that can effectively relieve T-cell suppression in tumor models, such as 13F3, have shown anti-inflammatory effects in sepsis models (42, 62, 90). This reversal may be related to the complex cytokine milieu and activation states of inflammatory cells in sepsis. In addition, VISTA plays multiple roles within receptor and ligand networks and may regulate immune-cell chemotaxis and migration. These functions could substantially shape the overall consequences of blockade. VISTA is also involved in maintaining Treg homeostasis. Its deficiency may impair Treg function and disrupt immune tolerance. Therefore, complete and sustained blockade in sepsis could destabilize tolerance, exacerbate inflammatory injury, and increase mortality risk (23). For clinical translation, a simple, continuous, and complete blockade strategy is unlikely to be appropriate. A more rational approach is to identify a suitable therapeutic window under close immune monitoring (91). Treatment should then be adjusted in an individualized and dynamic manner, guided by lymphocyte counts, HLA-DR expression, cytokine kinetics, and the risk of secondary infections (7, 9, 60, 92).

Different modes of modulation and intervention time points can lead to opposite outcomes. This likely reflects both the evolving immune trajectory of sepsis and the multifunctional nature of VISTA. These findings further suggest that VISTA may regulate T cells and macrophages in different directions, and that this divergence may depend on disease stage and microbial activity. Finally, the host response in sepsis is highly heterogeneous. Hyperinflammation, immunosuppression, and endothelial injury often coexist and interact. For this reason, a single target may be insufficient to restore immune balance. In sepsis patients with immunosuppressive phenotypes, combining VISTA blockades with immune-stimulating therapies, such as GM-CSF, IL-7, or PD-1 inhibitors, may provide synergistic effects and enhance immune function.

To directly interrogate the biphasic hypothesis advanced herein, we propose a focused experimental framework. Specifically, we recommend the adoption of a single, well-characterized CLP model in which VISTA function is modulated—through either blockade or agonism—at two pre-defined time points: an early window (2–6 hours post-CLP) corresponding to the hyperinflammatory phase, and a late window (48–72 hours post-CLP) corresponding to the immunosuppressive phase. Beyond survival as a primary endpoint, comprehensive immunological profiling should be undertaken, encompassing systemic cytokine signatures, T cell functionality (proliferative capacity and effector cytokine production), myeloid cell phenotype (notably HLA-DR expression and the expansion of myeloid-derived suppressor cells), and bacterial clearance kinetics. Such a design would permit a temporally resolved dissection of VISTA’s context-dependent contributions to sepsis pathophysiology.

## 5 Challenges and future perspectives of VISTA immunotherapy in sepsis

### 5.1 Challenges and future perspectives

Research on VISTA in sepsis is evolving from descriptive phenotypic associations toward mechanistic understanding and therapeutic development; however, several key challenges remain. The immune response during sepsis is highly dynamic, with substantial heterogeneity across disease stages and immune cell types (81). Current evidence suggests that in the acute phase, VISTA may be protective by limiting myeloid-driven inflammatory amplification, supporting Treg function, and maintaining mucosal homeostasis (22–24, 61, 62). However, the same pathway may also contribute to pathological immune tolerance and sepsis-associated immunosuppression (8, 59, 68). In addition, the expression patterns and functional roles of VISTA differ significantly among monocytes/macrophages, neutrophils, T cell subsets, and barrier-associated cells (23, 61, 68). This cellular heterogeneity increases the complexity of VISTA-targeted therapies, and highlights the urgent need to define VISTA functions across distinct immune contexts relevant to sepsis.

### 5.2 Complexity of tissue-specific immune activity

Compounding this challenge, immune status measured in peripheral blood does not reliably reflect immune activity within solid organs. The lung, gut, liver, and brain each possess unique tissue microenvironments and resident immune cell compositions (4, 7). Importantly, VISTA exhibits pH-dependent ligand interactions and is highly expressed by tissue-resident immune cells (30), meaning its regulatory effects may be pronounced in some tissues but negligible in others (57). Therefore, spatially resolved tissue immune profiling using samples such as bronchoalveolar lavage fluid, intestinal mucosa, brain tissue, and cerebrospinal fluid will be key to further elucidating the true *in vivo* role of VISTA in sepsis.

### 5.3 Fundamental biological and therapeutic design challenges

Although candidate ligands such as VSIG-3 and PSGL-1 have been identified, and pH-dependent binding has been characterized, their functional relevance *in vivo* within infectious inflammatory microenvironments remains insufficiently defined (30, 48). Downstream signaling pathways mediating VISTA’s bidirectional effects also require systematic investigation. In parallel, antibody epitope selection is a critical determinant of therapeutic outcome: antibodies targeting different VISTA epitopes may function as either antagonists or agonists. These fundamental biological issues will directly influence epitope selection, dosing strategies, and companion biomarker development. Given that sepsis progresses from a hyperinflammatory state to immune paralysis, future drug design must clearly define the clinical context in which blockade versus agonism is therapeutically appropriate (27, 39). In addition, tunable therapeutic approaches, including bispecific antibodies, may be required to adapt to the complex immune milieu of sepsis.

### 5.4 Limitations of preclinical models and need for clinical translation

At present, most studies on VISTA in sepsis rely on preclinical animal models, which have inherent limitations. Endotoxemia induced by LPS and polymicrobial infection induced by CLP exhibit substantially divergent immune activation kinetics. Furthermore, iARDS models combining hemorrhagic shock with infection align with the “two-hit” disease concept and may better mimic post-traumatic sepsis in humans (61, 62). These models also introduce additional variables that complicate result interpretation. Notably, clinical studies linking VISTA-related immune phenotypes to patient outcomes are lacking, and clinical trials targeting VISTA in sepsis have not yet been initiated. Importantly, either blockade or agonism of VISTA may produce opposite effects across different patient subgroups. Therefore, successful clinical translation will require biomarker-matched precision patient stratification, a framework for dynamic intervention, rigorous safety monitoring, and predefined stopping rules. An open-label, first-in-human, Phase 1 study (NCT02671955) designed to evaluate the safety, pharmacokinetics, and pharmacodynamics of JNJ-61610588, a fully human IgG1 kappa anti-VISTA monoclonal antibody, in patients with advanced cancer. The trial was registered but terminated early after enrolling only 12 subjects. Although a recent development indicates that ^89^Zr-labelled CI-8993 is now suitable for targeting and imaging VISTA expression in human trials (93), no results from the JNJ-61610588 study have been posted, and the study remains unpublished. It warrants further clinical trials that correlate VISTA expression on specific immune cell subsets (e.g., monocytes, T cells) with key clinical endpoints such as 28-day mortality, the development of secondary infections, progression to immune paralysis, and standardized assays to measure VISTA in clinical settings.

### 5.5 Human-mouse differences in VISTA biology and implications for clinical translation

A critical challenge in translating VISTA-targeted therapies from preclinical models to human sepsis patients lies in the fundamental biological differences between murine and human immune systems. While the majority of mechanistic insights into VISTA function have been derived from rodent models, emerging evidence reveals significant species-specific disparities in VISTA signaling, expression patterns, and functional outcomes that must be explicitly addressed to avoid erroneous clinical assumptions.

In murine models, VISTA is consistently characterized as a potent inhibitory immune checkpoint that suppresses T cell activation and restrains myeloid-driven inflammation. However, the work of Rogers et al. (49) has demonstrated that, in humans, VISTA can function as an activating receptor on monocytes, potentially promoting pro-inflammatory cytokine production rather than suppressing it. This finding has profound implications: if VISTA blockade in mice dampens inflammation (as observed with the 13F3 antibody in iARDS models), the same therapeutic strategy in humans might paradoxically attenuate a protective activating signal, leading to unintended immune suppression or altered inflammatory dynamics. This fundamental signaling dichotomy underscores that therapeutic strategies cannot be directly extrapolated from murine data and must be validated using human immune cells.

While VISTA is constitutively and highly expressed on myeloid lineage cells in both species, its regulation on T cell subsets during inflammation may differ substantially. In mice, VISTA expression is upregulated on CD4+ regulatory T cells (Tregs) following septic challenge, and its deficiency impairs early Treg maintenance and expansion, contributing to worse outcomes. However, the dynamics of VISTA expression on human CD4^+^ and CD8^+^ T cell subsets during sepsis, particularly their activation-induced regulation, co-expression with other checkpoint molecules, and susceptibility to functional modulation, remain largely uncharacterized (23). We emphasize that the functional consequences of VISTA ligation on human T cells, especially in the complex cytokine milieu of sepsis, require direct investigation in well-phenotyped patient cohorts before any clinical intervention can be rationally designed.

### 5.6 VISTA as a biomarker for patient stratification: current status and future needs

A central claim of this review is that VISTA expression could serve as an “actionable biomarker” for patient stratification to guide stage-specific immunotherapy in sepsis. However, this concept remains largely speculative, and a critical gap exists between the promising preclinical data and the clinical validation required for its implementation. This section outlines the current status of VISTA measurement, the scarcity of human sepsis data, and a roadmap for future research to translate this biomarker concept into clinical practice (**Table 2**, **Supplementary 1**).

**Table 2:** Clinical trials of VISTA from ClinicalTrials.gov (All enrolled participants were adult and older adult patients of both sexes).

NCT number	Status	Results	Conditions	Interventions	Phases	Type	Design	Primary completion date (Estimated)	Last update posted
NCT02812875	Completed	NO	Advanced Solid Tumors or Lymphomas	Drug: CA-170	Phases 1	interventional	Allocation: NA. Intervention Model: single group. Masking: none. Primary Purpose: treatment	2020/5/7	2020/6/26
	Study Title	A Study of CA-170 (Oral PD-L1, PD-L2 and VISTA Checkpoint Antagonist) in Patients with Advanced Tumors and Lymphomas							
	Brief Summary	CA-170 is a rationally designed and orally available, small molecule that directly targets the PD-L1/PD-L2, and VISTA immune checkpoints and results in activation of T cell proliferation and cytokine production. This is a multi-center, open-label, Phase 1 trial of orally.							
	Primary Outcome Measures	1. The number of patients with a dose-limiting toxicity (DLT) in the first treatment cycle, Approximately 24 months. 2. Maximum tolerated dose (MTD) of CA-170, Approximately 24 months|Recommended Phase 2 Dose (RP2D) of CA-170, Approximately 24 months.							
NCT02671955	Terminated	NO	Advanced Cancer	Drug: JNJ-61610588	Phases 1	interventional	Allocation: non randomized. Intervention Model: single group. Masking: none. Primary Purpose: treatment	2017/1/1	2018/3/27
	Study Title	A Study of Safety, Pharmacokinetics, Pharmacodynamics of JNJ-61610588 in Participants with Advanced Cancer							
	Brief Summary	This is a phase 1, open-label, multicenter dose-escalation study to determine the RP2D of CI 8993 for administration to patients with relapsed/refractory solid tumors by evaluating the safety and tolerability and characterizing the PK, PD, and anti-cancer activity of CI-8993 in this population.							
	Primary Outcome Measures	1. Frequency of Dose Limiting Toxicity (DLT), The Dose Limiting Toxicity (DLT) is based on adverse events and includes unacceptable hematologic toxicity, unacceptable non-hematologic toxicity of Grade 3 or higher, and treatment delay greater than 2 weeks., Approximately 2.5 years. 2. Number of Participants with Adverse Events (AEs) and Serious AEs, An adverse event (AE) is any untoward medical occurrence in a participant who received study drug without regard to possibility of causal relationship. A serious adverse event (SAE) is an AE resulting in any of the following outcomes or deemed significant for any other reason: death; initial or prolonged inpatient hospitalization; life-threatening experience (immediate risk of dying); persistent or significant disability/incapacity; congenital anomaly., Approximately 2.5 years. 3. Change From Baseline in Pharmacodynamic Blood Biomarkers- Total Blood Cell Counts, Standard hematology laboratory tests will be used to evaluate total blood cell counts in blood samples collected pre- and posttreatment., Approximately 2.5 years. 4. Change From Baseline in Pharmacodynamic Blood Biomarkers- Markers of Monocyte Activation, Flow cytometry will be used to evaluate markers of monocyte activation in blood samples collected pre- and posttreatment., Approximately 2.5 years. 5. Change From Baseline in Pharmacodynamic Blood Biomarkers- Markers of T Cell Activation, Flow cytometry will be used to evaluate markers of T cell activation in blood samples collected pre- and posttreatment., Approximately 2.5 years. 6. Change From Baseline in Pharmacodynamic Tissue Biomarkers- Protein Expression of VISTA, Pre- and posttreatment tissue samples will be stained by immunohistochemistry for protein expression of VISTA., Approximately 2.5 years. 7. Change From Baseline in Pharmacodynamic Tissue Biomarkers- Markers Associated with Immune Infiltrate Including CD3, CD4, CD8, Forkhead box P3, CD68, and PD-L1., Pre- and posttreatment tissue samples will be stained by immunohistochemistry for markers associated with immune infiltrate including CD3, CD4, CD8, forkhead box P3, CD68, and PD-L1., Approximately 2.5 years.							
NCT04475523	completed	NO	Solid Tumor	Drug: CI-8993	Phases 1	interventional	Allocation: NA. Intervention Model: single group. Masking: none. Primary Purpose: treatment	2023/5/19	2023/10/27
	Study Title	Phase 1 Study of CI-8993 Anti-VISTA Antibody in Patients with Advanced Solid Tumor Malignancies							
	Brief Summary	This is a phase 1, open-label, multicenter dose-escalation study to determine the RP2D of CI 8993 for administration to patients with relapsed/refractory solid tumors by evaluating the safety and tolerability and characterizing the PK, PD, and anti-cancer activity of CI-8993 in this population.							
	Primary Outcome Measures	1. To determine the maximum tolerated dose of CI-8993, The highest dose at a schedule, at which the DLT rate during the first cycle of this study (28 days from the first full dose) is< 33% in at least 6 patients., Approximately 2 years. 2. Determine the Recommended Phase 2 dose (RP2D), The RP2D will be a dose considered to be appropriately safe for a target phase 2 population and exhibit PK and PD characteristics that are favorable and considered likely to support clinical efficacy of CI-8993. The RP2D will be defined by the Safety Review Committee (SRC) based on PK, PD, safety, efficacy results in this study, as well as practical limitations., Approximately 2 years.							
NCT05082610	completed	NO	Cancer, Solid Tumor, Nonsmall Cell Lung Cancer, Triple Negative Breast Cancer, Malignant Neoplasm, Metastatic Cancer, Advanced Solid Tumor.	Drug: HMBD-002, and Pembrolizumab	Phases 1	interventional	Allocation: non randomized. Intervention Model: parallel. Masking: none. Primary Purpose: treatment	2024/11/5	2025/10/16
	Study Title	A Study of HMBD-002, a Monoclonal Antibody Targeting VISTA, as Monotherapy and Combined with Pembrolizumab, in Patients with Advanced Solid Tumors							
	Brief Summary	This is a phase 1/2, open-label, multi-center, first-in-human, two-stage (Part 1: dose escalation and Part 2: dose expansion) study evaluating multiple doses and schedules of intravenously (IV) administered HMBD-002, with or without pembrolizumab KEYTRUDA?, in patients with advanced solid tumors (i.e., locally advanced and unresectable, or metastatic)							
	Primary Outcome Measures	1. Dose-limiting Toxicity, The incidence of DLTs during the DLT assessment period., First 21 days of treatment. 2. Dose-Finding, Determination of the MTD or maximum tested dose, and the RP2D., Screening to 90 days from last dose. 3. Frequency and Severity of Adverse Events (AE), The incidences and percentages of patients experiencing AEs summarized by NCI CTCAE version 5.0 grade and by causality., Screening to 90 days from last dose.							
NCT05864144	Active not recruiting	NO	Cancer, Melanoma, Sarcoma, Advanced Cancer, Metastatic Cancer, Refractory Cancer, Nonsmall Cell Lung Cancer, Merkel Cell Carcinoma.	Drug: SNS-101 (anti-VISTA) and Cemiplimab	Phases1, Phases 2	interventional	Allocation: non randomized. Intervention Model: sequential. Masking: none. Primary Purpose: treatment	2027/6/1	2025/8/15
	Study Title	A Study of SNS-101 (Anti VISTA) Monotherapy and in Combination with Cemiplimab in Patients with Advanced Solid Tumors							
	Brief Summary	Phase 1/2 study to evaluate the safety, tolerability, pharmacokinetics, pharmacodynamics, and efficacy of SNS-101, a novel anti VISTA IgG1 monoclonal antibody as monotherapy or in combination with cemiplimab in patients with advanced solid tumors.							
	Primary Outcome Measures	1. Adverse Events - Part A & B, Incidence, nature and severity of treatment-related adverse events, Day 1 through 90 days after the last dose. 2. Determine the Recommended Phase 2 dose or maximum tolerated dose - Part A & B, Incidence and nature of dose-limiting toxicities, Approximately 15 months. 3. Objective Response Rate (ORR) - Part C, Measured by RECIST 1.1 and iRECIST, Day 1 through study completion (approximately 1 year).							
NCT06302556	unknown	NO	Lung Transplant Rejection	Diagnostic test: Immune checkpoints analysis, and Gene expression analysis	/	observational	Observational model: cohort. Time Perspective: other	2026/1/1	2024/3/8
	Study Title	The Role of Immune Checkpoints in Lung Transplant (ILTRA)							
	Brief Summary	The goal of this observational study is to learn about rejection in lung transplantation. The main question it aims to answer is: what is the role of immune checkpoints in lung transplantation? Participants will describe pathways of rejection in lung transplantation analyzing the immune checkpoints on explanted lungs as well as trans-bronchial biopsies.							
	Primary Outcome Measures	1.Lung-tissue immune-checkpoint profile and bronchoalveolar immune-cells mRNA signature in acute rejection after lung transplantation., Prevalence of immune checkpoint markers and gene-expression on leukocytes from transbronchial biopsies and bronchoalveolar lavage. Participants: cohort number 3., Twelve months after lung transplantation (prospective data). 2. Lung-tissue immune-checkpoint profile in chronic rejection after lung transplantation., Prevalence of immune checkpoint markers on leukocytes from explanted lung grafts and transbronchial biopsies. Participants: cohort number 1 and 2., Cohort 1: at re-transplantation for chronic lung allograft dysfunction (cross-sectional data). Cohort 2: from one to 36 months after lung transplantation (retrospective data).							
NCT06342661	recruiting	NO	Cerebrovascular Disease Small Vessel	Diagnostic test: Neuropsychological testing	/	observational	Observational Model: case control. Time Perspective: retrospective	2028/11/1	2024/4/2
	Study Title	Study On the Expression Level and Clinical Significance of VISTA in Patients with Vascular Cognitive Impairment							
	Brief Summary	1. Explore the expression level of immune checkpoint VISTA in peripheral blood and cerebrospinal fluid of patients with cardiovascular risk factors; 2. Discuss the correlation between different risk factors (mainly hypertension, diabetes, smoking, hyperlipidemia, hyperhomocysteinemia, etc.) and the expression level of immune checkpoint VISTA in peripheral blood and cerebrospinal fluid of patients with cerebrovascular diseases and immune-inflammatory related diseases of the nervous system; 3. Explore the correlation between different immune inflammatory factors (IL-1, IL-6, IL-10, INF-γ, TNF-α) and the expression level of immune checkpoint VISTA in peripheral blood and cerebrospinal fluid of patients; 4. Track and explore the dynamic changes of immune checkpoint VISTA in peripheral blood and cerebrospinal fluid of patients with cardiovascular risk factors in 3 months, 1 year, 3 years, and 5 years.							
	Primary Outcome Measures	1. Patient characteristics, 2023.03.01-2028.11.30. 2. VISTA expression in PBMCs in the VCI and control groups, 2023.03.01-2028.11.30.							

As of this writing, there are very few published studies that have systematically characterized VISTA expression dynamics in well-phenotyped human sepsis cohorts. The limited available evidence, primarily from small-scale observational studies, suggests that VISTA is upregulated on circulating monocytes in some septic patients, particularly those with evidence of immunosuppression (88). However, its correlation with disease severity (e.g., SOFA score), clinical stage (hyperinflammation vs. immune paralysis), or long-term outcomes (e.g., 28-day mortality, secondary infection rate) has not yet been established in any large, prospective cohort. We explicitly state that this absence of robust clinical data is a critical knowledge gap that must be addressed before VISTA can be considered a clinically actionable biomarker. The field currently lacks standardized assays, validated cut-off values, and a consensus on which specific cell subset or expression pattern is most informative for clinical decision-making.

Given the current state of evidence, it is impossible to define threshold levels or actionable VISTA phenotypes for patient stratification. To bridge this gap, we propose a structured roadmap for future research. Firstly, the highest priority is to conduct large-scale, multi-center prospective cohort studies that measure VISTA expression dynamics on key immune cell subsets (monocytes, T cells, MDSCs) using standardized, harmonized flow cytometry panels. Secondly, the VISTA expression data must be rigorously correlated with established clinical parameters (e.g., SOFA score, need for vasopressors, presence of secondary infections, 28-day and 90-day mortality) and validated immune status biomarkers (e.g., monocyte HLA-DR expression, absolute lymphocyte count, cytokine profiling). This will allow researchers to determine whether specific VISTA expression patterns are associated with distinct clinical trajectories and immune phenotypes. Thirdly, after such rigorous clinical validation, demonstrating that VISTA expression levels are independently associated with clinically meaningful outcomes and can identify patients likely to benefit from stage-specific VISTA modulation, can VISTA be considered a reliable companion biomarker for guiding immunotherapy.

### 5.7 Safety considerations and potential for combination therapies

Particular attention should be paid to clinically significant risks, including rebound inflammation, hemodynamic instability, and increased secondary infections (6, 9, 60). Finally, combination strategies targeting multiple immune checkpoints have a strong biological rationale in sepsis. As sepsis involves multifaceted immune dysregulation, targeting a single molecule may be insufficient to restore immune homeostasis. Combining VISTA blockade or modulation with other immunotherapies, such as PD-1/PD-L1 inhibitors or TLR agonists, may enhance therapeutic efficacy. However, such approaches must be pursued with caution, and preclinical validation is essential to avoid unexpected functional antagonism and harmful drug-drug interactions (94).

Overall, VISTA-targeted immunotherapy holds significant therapeutic potential for sepsis. Addressing these challenges, defining feasible interventional strategies, and refining priorities for future research will be critical to advancing the field toward clinical translation. Given the high morbidity and mortality of sepsis, and the inherent risks of immunomodulatory therapy, sustained mechanistic and clinical investigations of VISTA may provide novel therapeutic options and ultimately improve outcomes for septic patients.

## 6 Conclusions

In this review, we have synthesized the rapidly evolving understanding of VISTA as a critical, dual-function immune checkpoint molecule in sepsis pathophysiology. Our analysis reveals that VISTA is not a simple inhibitor or activator, but a context-dependent rheostat that calibrates immune responses across the sepsis continuum. The stage-specific, biphasic nature of VISTA’s role, protective in early hyperinflammation yet potentially detrimental in late immunosuppression, underscores the necessity for precision immunomodulation rather than blanket blockade or agonism. In conclusion, VISTA represents a paradigm shift in our understanding of sepsis immunopathology, moving from a model of simple immune dysregulation to one of context-dependent, stage-specific immune checkpoint control. The successful translation of VISTA-targeted therapies will depend on our ability to embrace this complexity, employing precision medicine approaches to restore, rather than override, the physiological homeostatic functions of this remarkable molecule.
